# High progesterone levels are associated with family history of premature coronary artery disease in young healthy adult men

**DOI:** 10.1371/journal.pone.0215302

**Published:** 2019-04-15

**Authors:** Tadeusz Osadnik, Natalia Pawlas, Kamila Osadnik, Kamil Bujak, Marta Góral, Mateusz Lejawa, Martyna Fronczek, Rafał Reguła, Hanna Czarnecka, Marcin Gawlita, Joanna Katarzyna Strzelczyk, Małgorzata Gonera, Marek Gierlotka, Lech Poloński, Mariusz Gąsior

**Affiliations:** 1 2nd Department of Cardiology and Angiology, Silesian Center for Heart Diseases, Zabrze, Poland; 2 Chair and Department of Pharmacology, School of Medicine with the Division of Dentistry in Zabrze, Medical University of Silesia, Katowice, Poland; 3 Institute of Occupational Medicine and Environmental Health, Sosnowiec, Poland; 4 3rd Department of Cardiology, School of Medicine with the Division of Dentistry in Zabrze, Medical University of Silesia in Katowice, Silesian Center for Heart Diseases, Zabrze, Poland; 5 Students’ Scientific Society, 3rd Department of Cardiology, School of Medicine with the Division of Dentistry in Zabrze, Medical University of Silesia in Katowice, Silesian Center for Heart Diseases, Zabrze, Poland; 6 Department of Medical and Molecular Biology, School of Medicine with the Division of Dentistry in Zabrze, Medical University of Silesia, Katowice, Poland; 7 Clinical Laboratory, Silesian Center for Heart Diseases, Zabrze, Poland; 8 Department of Environmental Medicine and Epidemiology, School of Medicine with the Division of Dentistry in Zabrze, Medical University of Silesia, Katowice, Poland; 9 Regional Specialized Hospital No. 4, Anesthesiology and Intensive Care Unit, Bytom, Poland; 10 Department of Cardiology, University Hospital in Opole, Faculty of Natural Sciences and Technology, Institute of Medicine, University of Opole, Opole, Poland; Florida International University, UNITED STATES

## Abstract

**Background & aims:**

The offspring of patients with premature coronary artery disease (P-CAD) are at higher risk for cardiovascular disease, compared with subjects without a family history (FH) of P-CAD. The increased risk for cardiovascular disease in subjects with FH of early-onset CAD results from unfavorable genetic variants as well as social, behavioral and environmental factors, which are more prevalent in this group. Previous studies have shown that specific sex hormone levels may be associated with the risk of cardiovascular disease. The aim of this study was to compare wide range of biochemical marker levels including i.e. the levels of luteinizing hormone (LH), follicle-stimulating hormone (FSH), progesterone, estradiol, testosterone and sex-hormone binding globulin (SHBG) between young healthy male adults with and without FH of P-CAD.

**Methods:**

The study group consisted of young healthy Polish male adults enrolled in a MAGNETIC case-control study, who were recruited between July 2015 and October 2017. The inclusion criteria were as follows: male sex, age ≥18 and ≤35 years old, FH of P-CAD (cases) or no P-CAD in first-degree relatives (controls). The comparison of continuous and categorical variables was performed using the Student’s t-test or the U-Mann–Whitney test, and Fisher’s exact test, respectively. The correlations between FSH, LH, testosterone, progesterone, SHBG and other laboratory parameters were assessed using the Spearman rank correlation test. Both univariable and multivariable logistic regression analyses were performed to assess the association between analyzed variables and FH of P-CAD.

**Results:**

A total of 411 subjects (223 cases and 188 controls) were included in the study. There was a higher prevalence of major cardiovascular risk factors in subjects with FH of P-CAD (smoking, higher total and LDL cholesterol levels, higher body mass index and lower HDL cholesterol level). Moreover, the offspring of patients with P-CAD had lower SHBG level, and higher LH and progesterone levels in the crude comparison, compared with individuals without FH of P-CAD. After adjustment for confounding variables, progesterone and LH were determined to be independently associated with FH of P-CAD.

**Conclusion:**

Progesterone and LH levels are significantly associated with FH of P-CAD, independent of traditional risk factors for CAD.

## Introduction

The frequency of cardiovascular disease before the age of 55 is lower in women. At the age of 55, the lifetime risk of cardiovascular disease for males and females is similar. Females are, however, more likely to suffer from cerebrovascular disease or develop heart failure, whereas males are more likely to suffer from coronary artery disease as an initial event [1]. These observed differences in the manifestation of atherosclerotic cardiovascular disease led to research focusing on the role of sex hormones in the pathophysiology of atherosclerosis. It has been postulated that the lower frequency of premature coronary artery disease (P-CAD) in premenopausal women compared to men is primarily due to the protective effect of female sex hormones [2,3]. Lower estrogen levels in females are associated with a higher risk of cardiovascular disease [2,3]. In contrast, other studies have shown that progestogens added to standard hormonal replacement therapy consisting of estrogens increased the risk of cardiovascular events in postmenopausal women [4,5]. It has also been shown that in men, lower levels of testosterone and estradiol were associated with a higher coronary artery calcium score in the Offspring and Third Generation Cohorts of the Framingham Heart Study [6].

P-CAD in first-degree relatives is a commonly known risk factor for coronary artery disease (CAD) [7]. This effect is due to both genetic and environmental factors [7]. As shown in previous studies, traditional risk factors for CAD, i.e., smoking, arterial hypertension, hypercholesterolemia, and abdominal obesity are also more prevalent in subjects with family history (FH) of P-CAD [8]. To the best of our knowledge, the relationship between gonadal and pituitary hormones and the FH of P-CAD has not been evaluated so far.

The aim of our study was to compare wide range of biochemical marker levels including i.a. pituitary hormones (follicle-stimulating hormone, FSH, and luteinizing hormone, LH) and sex steroids (testosterone, estradiol, progesterone) between young healthy adult men with and without FH of P-CAD.

## Methods

The following analysis is a part of the MAGNETIC (Metabolic and Genetic Profiling of Young Adults with and without a Family History of Premature Coronary Heart Disease) project. The MAGNETIC project is a case-control study that aims to analyze the classical and genetic risk factors of CAD in healthy young adults with and without FH of P-CAD. The study design of the MAGNETIC project has been described previously [9].

In brief, the study sample was recruited between July 2015 and October 2017. The inclusion criteria were as follows: male sex, age ≥18 and ≤35 years old, angiographically documented P-CAD (myocardial infarction, coronary artery bypass grafting, or percutaneous coronary intervention before the age of 55 in men and 65 in women) in first-degree relatives (test group) or no P-CAD in first-degree relatives (control group). The exclusion criteria for both groups were as follows: age <18 or >35 years, failure to provide informed consent, and acute or chronic diseases requiring pharmacotherapy. Subjects with a positive FH of P-CAD were recruited in the following two ways: 1) from healthy subjects aged 18 to 35 years who were asked to participate in a screening appointment at the Silesian Centre for Heart Disease, who provided documented proof of P-CAD history in their first-degree relatives, and 2) from the healthy offspring of patients who were hospitalized at our center between 2010 and 2017 due to P-CAD, who gave permission to be contacted once the treatment was completed. The control group was recruited from healthy subjects aged 18 to 35 years who were asked to participate in a screening appointment at our center, who were confirmed not to have FH of P-CAD. In total, 411 male Caucasian subjects met the inclusion criteria and were included in the study. Of these participants, 223 (54.3%) had first-degree relatives with documented P-CAD (test group), and 188 (43.7) subjects reported no FH of P-CAD (controls).

During the screening appointment, the recruited subjects, along with a qualified interviewer, filled out a detailed, standardized questionnaire encompassing sociodemographic information and questions regarding FH of cardiovascular diseases. Arterial blood pressure, weight and height were measured. Moreover, waist and hip circumference were measured using a measuring tape according to the recommendations of the World Health Organization [10]. Further details about the data collection procedures were described previously [9].

Peripheral blood was collected from each patient during the first recruitment visit between 7 am and 9 am., approx. 8 to 10 hours after the last meal. Fibrinogen content was determined in the blood collected in S-Monovette tubes with 3.1% sodium citrate (SarstedtAG&Co. KG. Germany) using a Siemens BCS XP analyzer (Siemens Healthcare, Germany). Biochemical and immunochemical measurements were determined in the patients’ serum using a Cobas 6000 analyzer (Roche Diagnostics. USA). Serum was obtained from the peripheral blood collected in S-Monovette tubes with clotting activator (SarstedtAG&Co. KG. Germany). The collected blood was centrifuged at 1500 rpm for 10 minutes at 4°C to obtain serum. The following concentration of the following components were measured using methods specified by the manufacturer: glucose, total cholesterol, high-density lipoprotein cholesterol (HDL-C), low density lipoprotein cholesterol (LDL-C), triglycerides, creatinine, uric acid, lipoprotein(a) (Lp(a)), apolipoprotein A1, apolipoprotein B, thyroid-stimulating hormone, FSH, LH, estradiol, progesterone, total testosterone, sex hormone binding globulin (SHBG), high-sensitivity C-reactive protein (hsCRP), total protein, and albumin. The HbA1C concentration in whole blood collected in S-Monovette tubes with EDTA was also measured using the Cobas 6000 analyzer. The test kits and catalog numbers are shown in S1 Table (Supporting Information). The percentage of high density lipoprotein (HDL%) was calculated according to the following formula: HDL-C level (mmol/L) divided by total cholesterol level (mmol/L) x 100.

Statistical analysis was performed using the computing environment R (R Development Core Team, 2005) [11]. The deviation from the normal distribution was investigated by analysis of the normal probability plots (Quantile-Quantile plots). The comparison of continuous variables was performed using the Student’s t-test or the U-Mann–Whitney test where appropriate. The categorical variables were assessed using Fisher’s exact test. The correlations between FSH, LH, testosterone, progesterone, SHBG and other laboratory parameters were assessed using the Spearman rank correlation test. We analyzed 27 variables for 411 patients. The 11097 element matrix contained 42 missing values (0.4%). The data on waist-to-hip ratio (WHR) were lacking for 10 patients, data on SBP and DBP measurements were lacking for 14 patients, information on smoking habits were missing for 2 patients, FSH was lacking for 1 patient and there was missing information on estradiol for 1 patient. We performed a univariable logistic regression analysis using complete observations to assess the association between analyzed variables and FH of P-CAD. Variables with p-values <0.35 in the univariable model were included in the multivariable analysis [12], except for total cholesterol, apolipoprotein B and HDL-C as they were significantly corelated with LDL and HDL%, and glucose because it was correlated with HbA1C. Next logistic regression with backward selection procedure was performed. Decision to retain variable in the model was based on Akaike information criterion. To minimize the impact of the missing data on the multivariable logistic regression analysis, we used missForest (R package missForest), a state of the art statistical method for mixed-type data imputation that utilizes the random forest model based on the non-missing variables in the dataset to predict the missing values. This process is repeated for all variables, and the process is iteratively repeated until a terminating criterion is attained [13].

Sample size of 188 patients in the control group and 223 patients in the test group (at α = 0.05 and β = 0.8), enabled us to detect difference of 27.8% of particular standard deviation value between analyzed groups. For example, we were able to detect difference of 0.139 units or less between means for variables with standard deviation of 0.5 or lower that is for variables such as: apolipoprotein B, apolipoprotein A1 [g/L], HDL-C [mmol/L], glucose [mmol/l], HbA1C [%], TSH [μlU/mL] and progesterone [ng/mL]. Power analysis was performed using PASS software (NCSS Statistical Software, Kaysville, Utah).

This study was conducted following the Declaration of Helsinki and good clinical practice guidelines. The study protocol has been approved by the Ethics Committee at Institute of Occupational Medicine and Environmental Health, Sosnowiec (Resolution no. 03/2013). Informed, written consent was obtained from all subjects enrolled in the study.

## Results

### Study group characteristics

**Fig 1:**
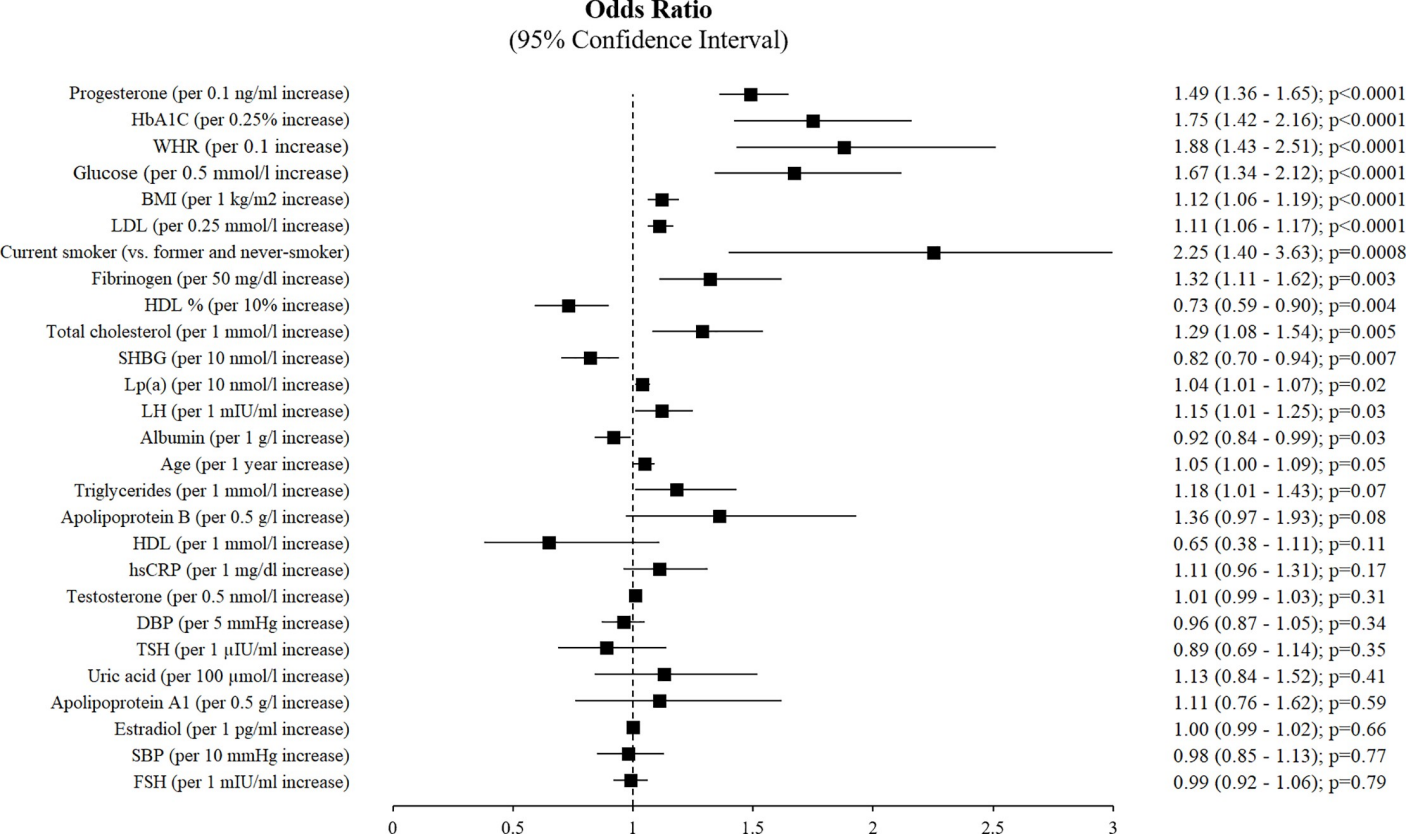
The association between analyzed variables and family history of premature coronary artery disease—univariable analysis.

Subjects with positive FH of P-CAD were slightly older (28.0 vs. 28.8 years, p = 0.03) and more frequently current smokers and obese, and they had a higher WHR compared with subjects without FH of P-CAD. Subjects with FH of P-CAD had higher levels of total cholesterol, LDL-C, apolipoprotein B, and lower levels of HDL-C (Table 1, Fig 1). In the test group, there were also significantly higher fibrinogen levels, a trend toward higher hsCRP, and lower albumin levels, compared with the control group (Table 1, Fig 1). Despite the fact that fasting glucose and HbA1c levels were within the normal range for all participants, the mean levels of glucose and HbA1c were significantly higher in subjects with positive FH (Table 1, Fig 1).

**Table 1:** The comparison of demographics, anthropometric factors and laboratory findings between young healthy male adults with and without family history of premature coronary artery disease.

Variable		Total sample	Family history of P-CAD		*p-value*
			With	Without	
N (%)		411 (100)	223 (54.3)	188 (45.7)	
Age [years]		28.5 ± 4.3	28.8 ± 4.5	28.0 ± 4.0	0.03
Current vs. never and former smoking (%)		103 (25.1)	71 (31.8)	32 (17.0)	0.0009
Physical examination					
	BMI [kg/m^2^]	25.9 ± 4.0	26.6 ± 4.0	24.9 ± 3.8	<0.0001
	WHR	0.89 ± 0.09	0.91 ± 0.08	0.87 ± 0.08	<0.0001
	SBP [mmHg]	131.2 ± 14.1	131.0 ± 13.8	131.4 ± 14.5	0.72
	DBP [mmHg]	80.0 ± 11.0	79.5 ± 11.5	80.6 ± 10.4	0.47
Laboratory test results					
	Cholesterol [mmol/L]	5.1 ± 1.2	5.2 ± 1.2	4.9 ± 1.1	0.005
	LDL-C [mmol/L]	3.2 ± 1.0	3.4 ± 1.0	3.0 ± 0.9	<0.0001
	Apolipoprotein B [g/L]	1.0 ± 0.3	1.01± 0.29	0.96 ± 0.29	0.08
	HDL-C [mmol/L]	1.4 ± 0.4	1.4 ± 0.4	1.5 ± 0.4	0.13
	Apolipoprotein A1 [g/L]	1.5 ± 0.3	1.5 ± 0.2	1.5 ± 0.3	0.27
	HDL [%] Triglycerides [mmol/L]	29.2 ± 9.5 1.4 ± 1.4	27.9 ± 9.4 1.5 ± 1.6	30.7 ± 9.5 1.2 ± 1.1	0.002 0.03
	Lp(a) [nmol/L]	42.8 ± 66.2	49.9 ± 72.7	34.5 ± 56.6	0.03
	Uric acid [μmol/L]	349.8 ± 66.5	352.3 ± 65.3	346.8 ± 67.8	0.41
	Albumin [g/L]	48.6 ± 2.5	48.3 ± 2.5	48.9 ± 2.5	0.03
	hsCRP [mg/dl]	1.3 ± 1.3	1.4 ± 1.4	1.2 ±1.2	0.08
	Glucose [mmol/l]	5.1 ± 0.5	5.2 ± 0.4	5.0 ± 0.5	<0.0001
	HbA1c [%]	5.05 ± 0.26	5.11 ± 0.25	4.97 ± 0.25	<0.0001
	Fibrinogen [mg/dL]	263.9 ± 58.0	271.7 ± 52.8	254.6 ± 62.5	<0.0001

Abbreviations: BMI–body mass index; DBP–diastolic blood pressure; HDL-C–high-density lipoprotein cholesterol; hsCRP–high-sensitivity C-reactive protein; LDL-C–low-density lipoprotein cholesterol; Lp(a)–lipoprotein(a); P-CAD–premature coronary artery disease; WHR–waist-to-hip ratio.

### Sex hormones

There were no significant differences in terms of testosterone, FSH and estradiol levels between the young healthy male subjects with and without FH of P-CAD. On the other hand, subjects with positive FH had lower SHBG levels and higher LH and progesterone levels, compared with the controls (Table 2).

**Table 2:** The comparison of hormone levels between young healthy male adults with and without family history of premature coronary artery disease.

Variable		Total sample	Family history of P-CAD		*p-value*
			With	Without	
Pituitary hormones					
	TSH [μlU/mL]	1.9 ± 0.8	1.9 ± 0.8	2.0 ± 0.8	0.32
	FSH [mlU/mL]	4.3 ± 2.7	4.3 ± 2.7	4.3 ± 2.8	0.92
	LH [mlU/mL]	4.7 ± 2.0	4.9 ± 2.1	4.5 ± 1.8	0.02
Gonadal hormones					
	Testosterone [nmol/L]	15.7 ± 5.8	16.0 ± 6.0	15.4 ± 5.6	0.52
	Estradiol [pg/mL]	21.4 ± 10.9	21.6 ± 9.8	21.1 ± 12.1	0.23
	Progesterone [ng/mL]	0.37 ± 0.31	0.5 ± 0.31	0.22 ± 0.23	<0.0001
Other					
	SHBG [nmol/L]	32.9 ± 15.7	30.9 ± 12.6	35.2 ± 18.5	0.007

Abbreviations: FSH–follicle-stimulating hormone; LH—luteinizing hormone; P-CAD–premature coronary artery disease; SHBG–sex hormone binding globulin; TSH—thyroid-stimulating hormone.

### The correlation of hormone levels with biomarkers associated with the risk of CAD

Both total testosterone and SHBG were inversely correlated with the following risk factors for CAD: BMI, WHR, total and LDL-C, apolipoprotein B, uric acid and fibrinogen levels. Additionally, a significant, positive correlation was found between testosterone levels and HDL-C, HDL%, and apolipoprotein A1 (Table 3). We did not detect any significant correlation between risk factors for CAD and FSH or LH, except for a weak correlation between age and LH (Table 3). Progesterone level was correlated with factors associated with insulin resistance, including WHR, fasting glucose and HbA1c levels. These correlations, although weak (r– 0.17, r– 0.14 and r– 0.24, respectively), were highly significant (p = 0.0005, p = 0.005 and p<0.0001, respectively), (Table 3).

**Table 3:** The correlations between hormone levels and age, anthropometric measurements and other laboratory findings.

Variable		Correlations with FSH	Correlations with LH	Correlations with estradiol	Correlations with progesterone	Correlations with testosterone	Correlation with SHBG
Age		0.19***	0.03	-0.14**	-0.004	-0.22****	-0.10*
Anthropometric measurements							
	BMI	-0.06	-0.09	-0.01	0.01	-0.41****	-0.46****
	WHR	0.08	-0.01	-0.04	0.18***	-0.26****	-0.29****
Other laboratory findings							
	Total cholesterol	0.06	-0.07	-0.15**	-0.02	-0.21****	-0.20****
	LDL-C	0.03	-0.09	-0.12*	0.03	-0.20****	-0.24****
	Apolipoprotein B	0.03	-0.09	-0.14**	-0.09	-0.26****	-0.26****
	HDL-C	0.04	0.03	0.07	0.04	0.26****	0.34****
	Apolipoprotein A1	0.08	0.05	-0.04	0.06	0.11*	0.13**
	HDL [%]	-0.01	0.06	0.15**	0.02	0.33****	0.39****
	Triglycerides	0.01	-0.05	-0.10*	0.02	-0.36****	-0.48****
	Lp(a)	0.01	0.07	-0.05	-0.03	-0.09	-0.09
	hsCRP	0.06	-0.01	-0.04	0.02	-0.30****	-0.29****
	Glucose	0.07	0.05	-0.17***	0.14**	-0.16**	-0.15**
	HbA1c	-0.07	-0.07	-0.01	0.24****	-0.21****	-0.21****
	Uric acid	0.02	-0.05	-0.01	-0.05	-0.28****	-0.36
	Fibrinogen	-0.01	-0.04	-0.06	0.07	-0.22****	-0.19****
	Albumin	-0.02	-0.03	-0.10*	-0.07	0.02	0.15**

p-value

*<0.05

** <0.01

*** <0.001

****<0.0001.

Abbreviations: BMI–body mass index; FSH–follicle-stimulating hormone; HDL-C–high-density lipoprotein cholesterol; hsCRP–high-sensitivity C-reactive protein; LDL-C–low-density lipoprotein cholesterol; LH—luteinizing hormone; Lp(a)–lipoprotein(a); SHBG–sex hormone binding globulin; WC–waist circumference; WHR–waist-to-hip ratio.

### Multivariable analysis of the association between analyzed parameters and FH of P-CAD

**Fig 2:**
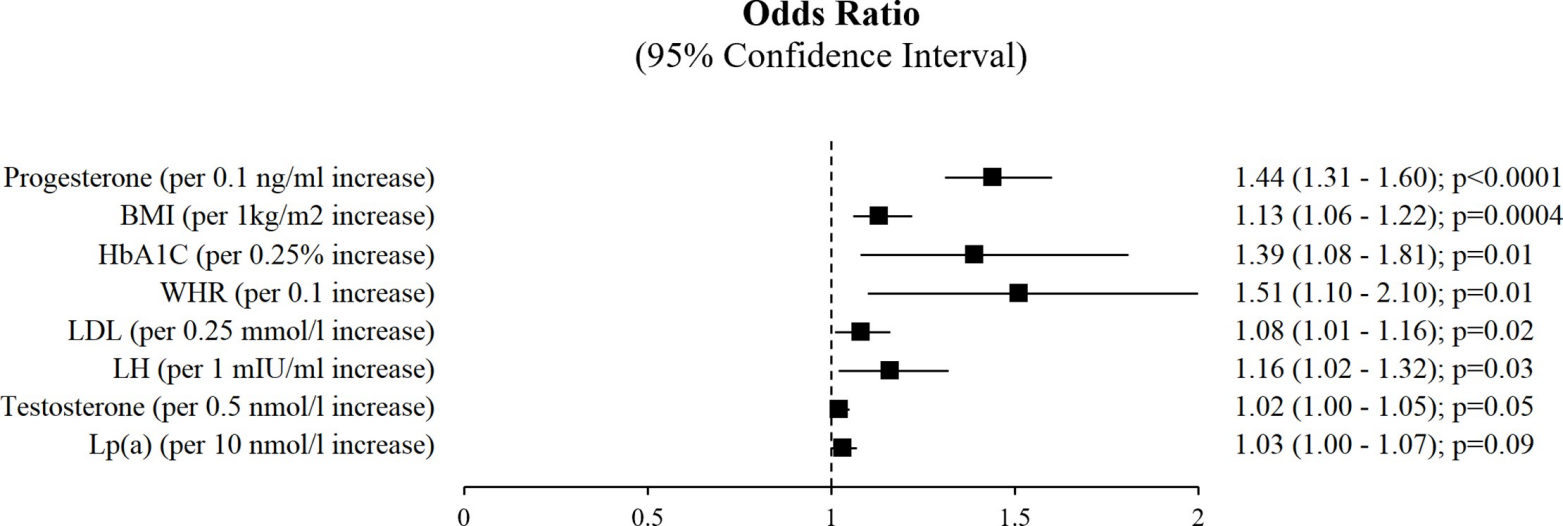
The association between analyzed variables and family history of premature coronary artery disease—multivariable analysis.

According to the multivariable model that was selected based on the information criteria, the factors independently associated with FH of P-CAD were progesterone, LH, BMI, WHR, HbA1c, and LDL-C. There was also a borderline significant trend regarding the relationship between testosterone and Lp(a) with positive FH of P-CAD (Fig 2).

## Discussion

Progesterone in men is synthesized in the testicles and adrenal glands, and its serum level, in contrast to that of women, does not alternate periodically or with age [14–17]. Higher levels of progesterone in the offspring of patients with P-CAD detected in the crude comparison, as well as the results of the multivariable analysis, indicate a potential association between progesterone levels and FH of P-CAD. To the best of our knowledge, no previous studies have analyzed the association between endogenous progesterone and cardiovascular disease in humans. The relationship between progesterone and the pathophysiology of CAD was the subject of a few experimental studies on animal models. In 1996, Hanke et al. demonstrated that in the rabbit model of atherosclerosis, progesterone therapy inhibited the beneficial effect of estrogen therapy on plaque size, likely by affecting arterial sex hormone receptors. Moreover, in the same study, therapy with progesterone alone was associated with considerably increased intimal thickening, compared with the castrated control rabbits [14]. Recently, Yang et al. demonstrated that progesterone induces the expression of macrophage CD36, a receptor for oxidized LDL that enhances foam cell formation. This effect was shown to be mediated by the transcription factor peroxisome proliferator-activated receptor γ [18]. Additionally, Wassmann et al. demonstrated that progesterone antagonizes the vasoprotective effect of estrogen by reducing the expression of extracellular superoxide dismutase and activating NADPH oxidase. Therefore, progesterone increases the production of reactive oxygen species in vascular smooth muscle cells [19].

In our study, progesterone levels were significantly correlated with WHR, fasting glucose and HbA1c, which are associated with insulin resistance. The role of progesterone in the regulation of glucose levels has been known since the 1980s [15] when it was demonstrated that progesterone increases glucose levels by inhibiting insulin-mediated glucose uptake and metabolism in adipocytes and skeletal muscles [15,20–23]. Most of the published studies analyzing the role of progesterone in the development of insulin resistance have concerned gestational diabetes; therefore, for obvious reasons, the results of these studies cannot be directly translated to men [23,24]. Nonetheless, insulin resistance is another potential mechanism through which progesterone levels may affect the risk of cardiovascular disease.

Testosterone is the central male sex hormone, which is produced primarily by Leydig cells in the testicles [25–27]. In serum, testosterone is primarily transported bound to proteins, and two-thirds of the bound testosterone is bound to SHBG [26,28]. The production of testosterone by Leydig cells is stimulated by LH, which is synthesized in the anterior pituitary gland [29]. Similar to previous studies, in the young, healthy, male subjects included in our analysis, testosterone and SHBG were significantly inversely correlated with fasting glucose, HbA1c, WHR, BMI, lipid parameters (total cholesterol, LDL-C, apolipoprotein B, triglycerides), as well as inflammatory biomarkers [25,28–31]. In observational studies it has been shown that men with lower testosterone levels are at higher risk for cardiovascular disease, and that subject on treatment with testosterone replacement therapy have a decreased risk of adverse cardiovascular events [28,32,33]. Nonetheless, observational studies have their limitations i.e. they are burdened with reverse causality bias [34]. Although some promising findings have originated from small randomized clinical trials (RCTs), which indicated an improvement of lipid parameters, fasting glucose and a decrease in body weight in men with hypogonadism treated with testosterone replacement therapy (TRT) [35,36], no beneficial effects of TRT in terms of cardiovascular disease have been demonstrated [36], with one RCT, discontinued because of the significantly higher incidence rate of adverse cardiovascular events in the TRT group [37]. In opposite to observational studies, mendelian randomization analyses demonstrated a potential, causative relationship between higher endogenous testosterone levels and unfavorable lipid profiles, type 2 diabetes, hypertension, and adverse cardiovascular events [34,38,39]. In our study, we have shown for the first time that in young healthy male adults, LH is independently associated with FH of P-CAD. Moreover, there was a borderline significant relationship between higher testosterone levels and FH of P-CAD in the multivariable analysis. Other factors independently associated with FH of P-CAD included a higher BMI and WHR as well as higher LDL and HbA1C levels, which was in agreement with previous studies and may be the result of both hereditary burden and environmental and lifestyle factors [8,40,41].

### Study limitations

Subject with FH of p-CAD, similarly as in other reports, were different form subjects without FH of p-CAD, with regard to BMI and other risk factors of atherosclerosis [8,40,41]. Taking into account the correlation between progesterone and factors associated with insulin resistance and to exclude the potential secondary effects of body weight differences, we addressed this issue in the multivariable analysis, which revealed that BMI was independently from progesterone associated with FH of p-CAD. Additionally, we compared progesterone levels between test group and controls in the following subgroups: normal body weight (BMI 18.5 to 24.9 kg/m^2^), overweight (BMI 25.0 to 29.9 kg/m^2^) and obesity (≥30.0 kg/m^2^). In all analyzed subgroups stratified by BMI, subjects with positive FH of P-CAD had significantly higher progesterone level compared with controls (S2 Table—Supporting Information).

The primary aim of our study was to compare wide range of biochemical marker levels between subjects with and without FH of P-CAD. Male and female sex hormones were measured in both sexes mainly because of logistic reasons, and we did not aim to test any specific hypothesis regarding sex hormones. Results regarding highly significant association between progesterone and FH of P-CAD, even after adjustment for classical risk factors, although surprising also to our team, are in line with the results of experimental studies. Considering the case-control design of our study, they should be interpreted as hypothesis-generating.

## Conclusions

Progesterone is significantly corelated with markers for insulin resistance in young healthy male adults. Both progesterone and LH levels are significantly associated with FH of P-CAD, independent of traditional risk factors for CAD.

## Supporting information

S1 Table List of reagent kits. (DOCX)

S2 Table The comparison of progesterone levels between subjects with and without family history of premature coronary artery disease, across BMI categories. (DOCX)

S1 File Dataset (raw data). Study dataset.
(XLSX)

S2 File Dataset (imputed data). Dataset with imputed values using random forest algorithm (used for multivariable analysis).
(XLSX)
