# Supplementary material for: High progesterone levels are associated with family history of premature coronary artery disease in young healthy adult men
Source: PLoS One. 2019 Apr 15;14(4):e0215302. doi: 10.1371/journal.pone.0215302 (PMC6464341; doi:10.1371/journal.pone.0215302)
Supplement: S1 Table — (DOCX) [file pone.0215302.s001.docx]

**S1 Table. List of reagent kits.**

| **Laboratory tests** | **Test name, company** | **Catalog number** |
| --- | --- | --- |
| Albumin | Albumin Gen.2, Roche Diagnostics, Indianapolis, IN, USA | 03183688122 |
| Apolipoprotein A1 | APOAT, Roche Diagnostics, Indianapolis, IN, USA | 03032566122 |
| Apolipoprotein B | APOBT, Roche Diagnostics, Indianapolis, IN, USA | 03032574122 |
| Total cholesterol | CHOL2, Roche Diagnostics, Indianapolis, IN, USA | 03039773190 |
| Creatinine | CREJ2, Roche Diagnostics, Indianapolis, IN, USA | 04810716190 |
| Estradiol | Elecsys Estradiol III, Roche Diagnostics, Indianapolis, IN, USA | 06656021190 |
| Follicle-stimulating hormone | Elecsys FSH, Roche Diagnostics, Indianapolis, IN, USA | 11775863122 |
| Glucose | GLUC3, Roche Diagnostics, Indianapolis, IN, USA | 04404483190 |
| HbA1C | A1C-3 (TQ), Roche Diagnostics, Indianapolis, IN, USA | 05336163190 |
| High density lipoprotein | HDLC3, Roche Diagnostics, Indianapolis, IN, USA | 04399803190 |
| High sensitive C-reactive protein | CRPLX, Roche Diagnostics, Indianapolis, IN, USA | 20764930322 |
| Lipoprotein Lp(a) | LPA2, Roche Diagnostics, Indianapolis, IN, USA | 05852625190 |
| Low density lipoprotein | LDL_C, Roche Diagnostics, Indianapolis, IN, USA | 03038866322 |
| Luteinizing hormone | Elecsys LH, Roche Diagnostics, Indianapolis, IN, USA | 11732234122 |
| Progesterone | Elecsys Progesterone III, Roche Diagnostics, Indianapolis, IN, USA | 70922539190 |
| Sex hormone binding globulin | Elecsys SHBG, Roche Diagnostics, Indianapolis, IN, USA | 03052001190 |
| Thyrotropin hormone | Elecsys TSH, Roche Diagnostics, Indianapolis, IN, USA | 11731459122 |
| Total testosterone | Elecsys Testosterone II, Roche Diagnostics, Indianapolis, IN, USA | 05200067190 |
| Triglycerides | TRIGL, Roche Diagnostics, Indianapolis, IN, USA | 20767107322 |
| Uric acid | UA2, Roche Diagnostics, Indianapolis, IN, USA | 03183807190 |
