# Supplementary material for: High progesterone levels are associated with family history of premature coronary artery disease in young healthy adult men
Source: PLoS One. 2019 Apr 15;14(4):e0215302. doi: 10.1371/journal.pone.0215302 (PMC6464341; doi:10.1371/journal.pone.0215302)
Supplement: S2 Table — (DOCX) [file pone.0215302.s002.docx]

**S2 Table. The comparison of progesterone levels between subjects with and without family history of premature coronary artery disease, across BMI categories.**

| **BMI category** | **Progesterone level [ng/mL] and family history of P-CAD** | | ***p value*** |
| --- | --- | --- | --- |
|  | **With FH of P-CAD** | **Without FH of P-CAD** |  |
| Normal body weight (BMI 18.5-24.9 kg/m^2^) | 0.55±0.34  (n=89) | 0.22±0.23  (n=101) | <0.0001 |
| Overweight (BMI 25.0-29.9 kg/m^2^) | 0.49±0.31  (n=99) | 0.2±0.23  (n=75) | <0.0001 |
| Obesity (≥30.0 kg/m^2^) | 0.41±0.25  (n=38) | 0.25±0.2  (n=15) | 0.02 |

Abbreviations: BMI – body mass index; FH – family history; P-CAD – premature coronary artery disease.
